# Cross-Modal Search for Social Networks via Adversarial Learning

**DOI:** 10.1155/2020/7834953

**Published:** 2020-07-11

**Authors:** Nan Zhou, Junping Du, Zhe Xue, Chong Liu, Jinxuan Li

**Affiliations:** Beijing Key Lab of Intelligent Telecommunication Software and Multimedia, School of Computer Science, Beijing University of Posts and Telecommunications, 100876 Beijing, China

## Abstract

Cross-modal search has become a research hotspot in the recent years. In contrast to traditional cross-modal search, social network cross-modal information search is restricted by data quality for arbitrary text and low-resolution visual features. In addition, the semantic sparseness of cross-modal data from social networks results in the text and visual modalities misleading each other. In this paper, we propose a cross-modal search method for social network data that capitalizes on adversarial learning (cross-modal search with adversarial learning: CMSAL). We adopt self-attention-based neural networks to generate modality-oriented representations for further intermodal correlation learning. A search module is implemented based on adversarial learning, through which the discriminator is designed to measure the distribution of generated features from intramodal and intramodal perspectives. Experiments on real-word datasets from Sina Weibo and Wikipedia, which have similar properties to social networks, show that the proposed method outperforms the state-of-the-art cross-modal search methods.

## 1. Introduction

With the rapid development of mobile networks and “we media” [1], cross-modal information search [2] has become a research hotspot. Users publish multimedia information on social network platforms such as Weibo and Twitter, where public opinion is expressed through natural language and visual information. Cross-modal information search meets users' needs for data diversity, especially on social networks. Various types of topics (e.g., news, tips, and stories) occur in multimedia forms on social networks, conveying valuable information for various users, including common people, companies, and regulators. The most direct way to fulfill users' diversified information needs is to maximally mine the resemblance and correlations of the information and present the content relevant to users' queries [3, 4]. However, cross-modal correlation analysis faces the basic challenge of bridging the heterogeneity gap [5, 6] between different media, which is also a key issue for cross-modal search.

Bridging the heterogeneity gap in multimodal data, which feature different statistical characteristics, is the major issue in analyzing and processing multimodal datasets with intelligent technologies [7]. In general, some current research addresses the problem by constructing multiple nonlinear transformations [8] to build a common semantic subspace for multimodal data through deep learning [9]. With the subspace, the nonlinear transformations are learned to generate feature representations for correlation maximization [10]. The representative classical methods are canonical correlation analysis (CCA) [11] and variants such as deep CCA (DCCA) [12]. With the development of tabular learning and deep learning research, such strategic methods have gradually been divided into two groups: real-valued representations and binary-valued representations [13]. Other works focus on selecting relevant features that are, then, adopted to construct correlations from multimodal features to achieve cross-modal search through feature selection and matching [14, 15]. The methods used according to this strategy are designed to discover dense feature clusters with high similarity learned by algorithms for cross-modal data [16].

In addition, the semantic sparseness of cross-modal data from social networks results in misleading content in both the textual and visual modalities. Cross-modal data on social networks present characteristics that reflect many aspects of real-world events in quality-restricted forms [14]. The massive quantity of cross-modal data on social networks provides an opportunity to uncover relations between events and discover additional content related to the target event in a variety of media. The forms and characteristics of social network cross-modal data require many details of features such as local correlations to be mined and learned by intelligent algorithms. To overcome the semantic sparseness of cross-modal data from social networks, we adopt self-attention [17] to discover the differential importance of local semantic features according to the target topic throughout the global representation sensors. Self-attention can be used to assign weight values for different items in feature sequences to perceive significance. Li et al. [18] proposed a positional self-attention with contention (PSAC) architecture to capture long-range dependencies and position information. Through the application of self-attention to perceive significance, PSAC significantly outperforms its predecessor. Gao et al. [19] presented hierarchical LSTMs with an adaptive attention method to perceive the spatial-temporal attention for visual regions or frames to predict related words. This method with adaptive attention outperforms the previous state-of-the-art methods.

In this paper, we propose a cross-modal search method for social network data that capitalizes on adversarial learning. In addition, we adopt self-attention-based neural networks to generate modality-oriented representations for further inter-modal correlation learning. A search module is implemented based on adversarial learning, through which the discriminator is designed to measure the distribution of generated features from intramodal and intramodal perspectives. The discrimination is deployed as a compound neural network whose parameters are optimized under union losses following the adversarial learning mechanism to generate the most appropriate representations of cross-modal data features. The contributions of the paper are summarized as follows.

- We propose a supervised cross-modal adversarial learning method integrated with self-attention. The method generates cross-modal representations following the original modality and topic label distributions from the perspective of social network data characteristics under the mechanism of self-attention.
- The proposed method incorporates local semantic features distributed as word groups in texts and blocks in images to maximize the cross-modal correlations based on adversarial learning.
- The part of the adversarial learning component in the designed adversarial learning framework is used effectively to rank the search results.

The unstandardized writing conventions of user-generated text and the frequently low quality of user-submitted images submitted on social networks result in semantic sparseness. Semantic sparseness is the main obstacle to cross-modal information search in social networks based on global semantic features. Our proposed method, cross-modal search with adversarial learning (CMSAL), integrates self-attention to explore local semantic features expressing key semantic features of the target topics. Words (in text) and pixel blocks (in images) conveying target topics are the local semantic features to be explored and mined. The generated representations integrated with the local semantic features constitute the semantic space for social network cross-modal information search. The designed maximum losses are optimized based on adversarial learning to promote the efficiency of the generated representations for cross-modal search. The learning method is trained iteratively with the representation-generating process from intramodal and intermodal perspectives. In classical generative adversarial networks (GANs) [20], the optimal discriminator is useless in most cases [21]. We reused the optimal intermodal and intramodal restriction to provide ranked search results based on distribution measures. In contrast to the existing methods, this paper takes the semantic sparseness of social network content into consideration for the specific task of cross-modal information search.

## 2. Related Works

### 2.1. Social Network Cross-Modal Search

With the development of information and mobile networks, social network platforms are becoming the most important source for multimedia data [22]. Cross-modal search strategies on social networks can be classified into two main groups: common semantic subspace learning and feature selection and matching. For multimodal data from social networks conveying more information [23], intelligent technologies are needed to excavate latent correlations within massive and complex cross-modal datasets from social networks. Cai et al. [24] proposed a joint topic model to track and search target social information based on cross-modal feature sequence analysis and learning. Fang et al. [25] proposed a data transformation method to handle heterogeneous data for cross-modal event analysis and searches in social networks. Qing et al. [26] proposed an event and content search method based on automatic identification and tracking from a large amount of cross-modal data from social networks. Lee et al. [27] provided a common search framework for online social network hotspot events. The method normalizes the data content of different media based on the graph-based algorithm combination sorting event list for content normalization. It unifies the stream-based media data and the registration-based cross-media data, which realizes the cross-media search for the target event. Zhang et al. [28] studied the hierarchical information quad-tree index structure based on spatiotemporal characteristics, including temporal proximity, spatial proximity, and visual relevance. The method is also used to solve cross-modal search problems in social networks. Deng et al. [29] proposed a deep hash network based on triplets for cross-modal retrieval of social networks. The method uses a triple label to describe the relative relationship between the three instances as a supervisor to capture a more general semantic correlation between cross-modal instances.

Social network cross-modal search is related to the traditional cross-modal search on multimedia representation extraction and correlation analysis. Furthermore, cross-modal contents from social networks need to pay attention to global and local semantic associations in semantic sparseness, which is determined by the characteristics of the social network data. The emergence of GANs [20] provides a series of methods for semantic extractions and representations under sparse semantic conditions that are gradually applied to the field of cross-media search.

### 2.2. Adversarial Learning Cross-Modal Search

Recently, GANs [20] have been widely used because of their ability to learn and process visual and sequenced features. A series of approaches have been proposed to reduce the gap between different modalities based on adversarial learning of the statistical characteristics of the transformed features. Following this strategy, He et al. [30] introduced a cross-modal retrieval method based on unsupervised adversarial learning. The method constructed an adversarial learning feature transformation for the statistical properties on cross-modal search. Peng et al. [5] proposed a method for common cross-modal representation based on GAN. Through well-learned cross-modality representations, many applications such as cross-modal similarity matching can be conducted. Gu et al. [4] provided a GAN-based method incorporating corporate generative models into cross-modality embedding for cross-modal search. The method encouraged the textual features as the basis to generate an image similar to the ground truth, and vice versa for images to texts. Shang et al. [31] proposed a dictionary learning-based cross-modal search method. The method used a dictionary learned as a feature a reconstructor, cooperating with adversarial learning to mine cross-modality statistical characteristics. Wen et al. [32] proposed a cross-modal search method based on similarity transferring. The method uses adversarial learning to build a semantic structure in the common representation subspace for preserving the semantic structure between unpaired items across different modalities. Wang et al. [33] proposed an adversarial learning retrieval method that imposed triplet constraints for feature generation to minimize the heterogeneous gap of cross-modal data with the same semantic labels. The greatest advantage of adversarial learning is cross-modal synthesis. Gao et al. [34] presented a method named the perceptual pyramid adversarial network (PPAN) to synthesize photorealistic images and texts based on adversarial learning. The method is composed of a generator optimized with perceptual loss to obtain diverse images and a discriminator for multiple purposes, such as semantic consistency, image fidelity, and class invariance.

For other strategies, deep quantization and deep hashing based on adversarial learning are also used for cross-modal search. Yang et al. [35] proposed a method known as shared predictive deep quantization (SPDQ). In this method, a shared semantic subspace is defined for cross-modal features. The method builds a joint deep network architecture to exploit compact cross-modal representations. The method preserves intramodal and intermodal similarities in an efficient way. Deep hashing also follows the strategy to learn compact binary code for cross-modal similarity computation efficiency. Li et al. [36] presented a self-supervised adversarial hashing (SSAH) method. The method learns the high-dimensional features and hash codes for cross-modality information through two adversarial networks. The search similarity is maximized according to the semantic relevance in a highly computationally efficient manner.

In contrast to traditional methods of latent semantic subspace learning [37], cross-modal search based on GAN or adversarial learning takes advantage of the capacity for feature distribution construction and discrimination learning [33]. There are also many methods that adopt adversarial learning for hashing to realize cross-modal search [38, 39]. These methods convert the matching problem in cross-modal search to the Hamming distance calculation based on the multimedia effective binary representation. Such a calculation strategy improves the matching efficiency of cross-modal search. However, in the construction of binary representations, some semantic features of the original multimedia are lost. The proposed method in this paper focuses on local semantic feature extraction based on self-attention [17] and adversarial learning [20] to solve the problem of minimizing the heterogeneity gap for cross-modal data with the same semantic labels.

## 3. The Proposed CMSAL Method

### 3.1. Problem Definition

In general, we define cross-modal data as *P*={*C*_1_, *C*_2_,…, *C*_*d*_}, 1 ≤ *d* ≤ *D*, meaning that there are *D* topics in the data domain on the amount. For each topic, related contents are expressed in the form of text and images as *C*_*d*_={*t*_1_, *t*_2_,…, *t*_*m*_, *v*_1_, *v*_2_,…, *v*_*n*_*|l*_*d*_} (1 ≤ *m* ≤ *M*, 1 ≤ *n* ≤ *N*). In each topic, there are *M* text instances and *N* image instances conveying the related semantic information to *C*_*d*_ labeled by *l*_*d*_. There are some special cases for (*M* ≥ 1, *N* = 0) and (*M* *=* 0, *N* ≥ 1), in which the problem degenerates into the unimodal case. Another case is (*M* = 1, *N* = 1). In this case, the situation agrees with most definitions in current works.

Raw text and images are preprocessed into representation features by word embedding [40] and VGGNet [41], according to the modality. The presentation features for texts and images are interfaces for further complex computing in the learning procedure. Let **X**_*d*_={**x**_*t*_^*d*,1^, **x**_*t*_^*d*,2^,…, **x**_*t*_^*d*,*m*^, **x**_*v*_^*d*,1^, **x**_*v*_^*d*,2^,…, **x**_*v*_^*d*,*n*^*| ***y**_*d*_} be the collection of cross-modal original features (word embedding features for text and CNN features for images) with the one-hot label vector **y**_*d*_ for topic *d*, in which **x**_*t*_^*d*,*m*^ represents the word embedding feature for the *m-*th text entry under topic *d*.

**Figure 1:**
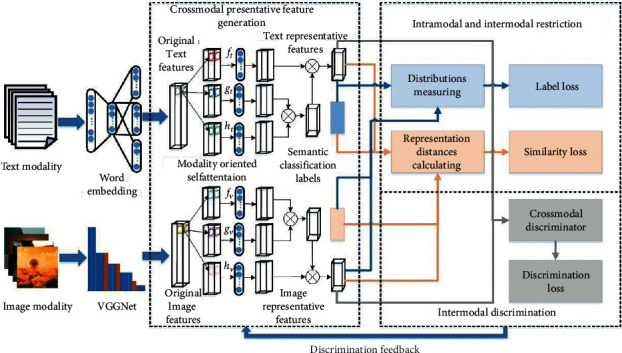
The architecture of the proposed CMSAL method.

For further correlation maximization learning, the presentation features are explored to extract local features that are sensitive to modality characteristics. The features convey the same semantics in word groups and image blocks represented as **b**_*t*_^*d*,*k*^, meaning the *k*-th block word feature, which is the same as **b**_*v*_^*d*,*k*^ with *K* blocks as an empirical value. The extraction process is defined as *S*_*t*_^*d*^(**x**_*t*_^*d*^; *θ*_*t*_). **x**_*t*_^*d*^={**b**_*t*_^*d*,1^**b**_*t*_^*d*,2^,…,**b**_*t*_^*d*,*k*^} shortened as *S*_*t*_^*d*^ for text representation features with the parameters of *θ*_*t*_, while *S*_*v*_^*d*^(**x**_*v*_^*d*^; *θ*_*v*_) · **x**_*v*_^*d*^={**b**_*v*_^*d*,1^**b**_*v*_^*d*,2^,…,**b**_*v*_^*d*,*k*^} shortened as *S*_*v*_^*d*^) for image features with the parameters of *θ*_*v*_. *S*_*t*_^*d*^ and *S*_*v*_^*d*^ are the generation processes interacting with the discriminator to optimize parameters jointly by adversarial learning. A restriction is designed to measure the distribution of *S*_*t*_^*d*^ and *S*_*v*_^*d*^ from intramodal and intermodal aspects to guide the generation. *S*_*t*_^*d*^ and *S*_*v*_^*d*^ output more appropriate representation features by episodes. The general framework of the proposed method is illustrated in Figure 1.

### 3.2. Constructions of Cross-Modal Representation Feature Generation

Cross-modal representation feature generation is conducted to explore the local semantic relationships between features from different modalities and reconstruct the representations to reflect the relationships in computational matrixes. The procedure is designed under a supervised representation learning mechanism in which self-attention is adopted. Taking text modality as an example, *f*_*t*_, *g*_*t*_, and *h*_*t*_ are the functions to transform the original features (word features for text in fixed-size blocks) into a subspace as follows:

(1) $$\begin{array}{c} f_{t}\left(b_{t}^{d,k}\right)=w_{t}^{f}b_{t}^{d,k},\\ g_{t}\left(b_{t}^{d,k}\right)=w_{t}^{g}b_{t}^{d,k},\\ h_{t}\left(b_{t}^{d,k}\right)=w_{t}^{h}b_{t}^{d,k}, \end{array}$$

where **b**_*t*_^*d*,*k*^ means the *k*-th text block word embedding feature of a text document on topic *d*. **w**_*t*_^*f*^, **w**_*t*_^*g*^, and **w**_*t*_^*h*^ are the parameter vectors of *f*_*t*_, *g*_*t*_, and *h*_*t*_. Similarly, *f*_*v*_, *g*_*v*_, and *h*_*v*_ are the corresponding functions for the image modality with the parameter vectors **w**_*v*_^*f*^, **w**_*v*_^*g*^, and **w**_*v*_^*h*^.

The original features of the two modalities are cut into fixed-size blocks. In general, we cut the original feature into *K* blocks. The blocks of original text features are composed of word vectors, while the blocks of original image features cover the CNN features of pixels. For example, the attention between the *i*-th and the *j*-th blocks is calculated as follows:

(2) $$\begin{array}{c} \beta_{t}^{d,i,j}=\frac{\exp\left(f_{t}{\left(b_{t}^{d,i}\right)}^{T}g_{t}\left(b_{t}^{d,j}\right)\right)}{\sum_{j=1}^{K}\exp\left(f_{t}{\left(b_{t}^{d,i}\right)}^{T}g_{t}\left(b_{t}^{d,j}\right)\right)}, \end{array}$$

where *β*_*t*_^*d*,*i*,*j*^ indicates the model attention parameter related to the *j*-th feature block when generating the representation features of the *i*-th block in the specific word embedding feature of the corresponding text on topic *d*. Similar to image modality, *β*_*v*_^*d*,*i*,*j*^ is used for images in CNN feature blocks. For the *i*-th block of a specific text piece of content, the representation features can be presented as follows:

(3) $$\begin{array}{c} o_{t}^{j}=\sum_{j=1}^{K}\beta_{t}^{d,i,j}h\left(b_{t}^{d,j}\right). \end{array}$$

The representation features of a whole text about the topic *d* can be presented as *S*_*t*_^*d*^={**o**_*t*_^1^, **o**_*t*_^2^,…, **o**_*t*_^*k*^}, which is the same as *S*_*t*_^*d*^={**o**_*v*_^1^, **o**_*v*_^2^,…, **o**_*v*_^*K*^}. It is regarded as a global semantic representation. The value of *K* is a hyperparameter determined by experiences and data contexts. In the experiment, we set the value of *K* according to the corresponding original cross-modality features. Otherwise, the value of *K* also determines the sizes of **w**_*t*_^*f*^ (**w**_*v*_^*f*^), **w**_*t*_^*g*^ (**w**_*v*_^*g*^), and **w**_*t*_^*h*^ (**w**_*v*_^*h*^) as parameters. However, it will have little impact on the actual representations through cross-modal presentative feature generation.

### 3.3. Learning Metric for the Proposed Method

In this section, we propose the generation and discrimination losses to train the proposed CMSAL. The generation loss guides the representation features generation and consists of a label loss and a similarity loss. The label loss aims to minimize the distribution difference between the representation features and corresponding topic semantic labels. The similarity loss is used to minimize the distance among the intermodal samples about the same topic. These two loss terms are defined as the generation loss for guiding the representation features generating procedure. The discrimination loss is defined to distinguish modalities. The multiple losses are collaborated into a minimax loss to optimize the generation of representation features for appropriate cross-modal search features.

#### 3.3.1. The Generation Loss

The generation loss is decomposed into two loss terms: the label loss and the similarity loss. The label loss ensures that the distributions generated representation features following those of semantic topics. The loss is presented as

(4) $$\begin{array}{c} L_{\text{label}}=-\left(\frac{1}{M}\sum_{i=1}^{M}y_{t}^{i}\log\hat{t}\left(S_{t}\left(x_{t}^{i};\theta_{t}\right)\right)+\frac{1}{N}\sum_{j=1}^{N}y_{v}^{j}\log\hat{t}\left(S_{v}\left(x_{v}^{j};\theta_{v}\right)\right)\right),\;y_{t}^{i}=y_{v}^{j}\text{ for }i=j, \end{array}$$

where **y**_*t*_^*i*^ and **y**_*v*_^*j*^ are the topic labels for corresponding features in the form of a one-hot vector. The symbol $\hat{t}$ is the function to predict topic probability distribution for each text or image term of the representation features. *M* and *N* are the amounts of the original features for text and images, respectively. As described in Section 3.1, we conduct the collection based on *M* = *N* for a clear expression and thinking. Therefore, equation (4) can be further expressed as follows:

(5) $$\begin{array}{c} L_{\text{label}}=-\frac{1}{M}\sum_{i=1}^{M}\left(y_{v}^{i}\log\hat{t}\left(S_{v}\left(x_{v}^{i};\theta_{v}\right)\right)+y_{t}^{i}\log\hat{t}\left(S_{t}\left(x_{t}^{i};\theta_{t}\right)\right)\right),y_{t}^{i}=y_{v}^{i}. \end{array}$$

The label loss guides the training of the parameters of ***θ***_*t*_ and *θ*_*v*_ to generate representation features following the topic distribution of corresponding samples. The label loss is the intramodal loss used to maintain the intramodal data correlations. Based on the premise of *M* = *N*, the similarity loss is defined as follows:

(6) $$\begin{array}{c} L_{\text{similarity}}=\frac{1}{MN}\sum_{i=1}^{M}\sum_{j=1}^{N}{\left({\left\|y_{t}^{i}-y_{v}^{j}\right\|}_{2}-{\left\|S_{t}\left(x_{t}^{i};\theta_{t}\right)-S_{v}\left(x_{v}^{j};\theta_{v}\right)\right\|}_{2}\right)}^{2}. \end{array}$$

The similarity loss acts as the intermodal loss to maximize correlations between cross-modal samples with the same topic distribution by closing the distance difference of representation features and topic labels.

The losses presented in equations (5) and (6) are the basics to guide representation feature generation by supervised learning for adjusting the parameters of the networks. As parts of the generation loss, the label loss and the similarity loss are integrated by weighted summation presented as equation (9).

(7) $$\begin{array}{c} L_{\text{generation}}=\alpha L_{\text{label}}+\beta L_{\text{similarity}}, \end{array}$$

where *α* and *β* represent the contribution weights of the corresponding deviation values to the loss function, through which the optimization of generation loss is directly affected by the two empirical values.

#### 3.3.2. The Discrimination Loss

The discrimination of the method is the key component to realize cross-modal adversarial learning. It aims to discriminate the modalities for the constructions of representation features about the same topic. We define the discrimination loss as follows:

(8) $$\begin{array}{c} L_{\text{discrimination}}=-\frac{1}{M}\sum_{i=1}^{M}m_{i}\left(\log\hat{p}\left(S_{v}\left(x_{v}^{i}\right);\theta_{p}\right)+\log\left(1-\hat{p}\left(S_{t}\left(x_{t}^{i}\right);\theta_{p}\right)\right)\right), \end{array}$$

where *m*_*i*_ is the modality label as a one-hot vector and $\hat{p}$ aims to map the generated representation features into the modality discrimination space under the parameter ***θ***_*p*_. Different from the generation loss, the discrimination loss promotes representation feature generation indirectly. The generator will output more appropriate representation features by parameter optimization and adversarial learning with a discriminator.

### 3.4. The Adversarial Training Procedure

To ensure the correlation maximum of cross-modal representation features for the same topic distribution, cross-modal representation feature generation and intermodal discrimination interact with adversarial learning. We construct the minimax game [20] as follows:

(9) $$\begin{array}{c} {\bar{\theta }}_{t},{\bar{\theta }}_{v}=\underset{\theta_{t},\theta_{v},\theta_{p}}{argmin}\left(L_{\text{generation}}\left(\theta_{t},\theta_{v}\right)-L_{\text{discrimination}}\left(\theta_{p}\right)\right),\\ {\bar{\theta }}_{p}=\underset{\theta_{p}}{argmax}\left(L_{\text{generation}}\left(\theta_{t},\theta_{v}\right)-L_{\text{discrimination}}\left(\theta_{p}\right)\right), \end{array}$$

where ${\bar{\theta }}_{t}$, ${\bar{\theta }}_{v}$, and ${\bar{\theta }}_{p}$ are optimized values for the joint losses. The minimax game will minimize generation loss and maximize the discrimination loss. The generation loss is going to construct cross-modal representation features to maximize relationships for the same semantic topic distribution. The discrimination loss will distinguish modality discrepancies. The parameters ***θ***_*p*_ are fixed for optimizing ***θ***_*t*_ and *θ*_*v*_ during the minimization procedure, while ***θ***_*t*_ and *θ*_*v*_ are fixed for optimizing ***θ***_*p*_ during the maximization procedure.

### 3.5. The Matching Similarity for CMSAL

Based on the optimized parameters, the cross-modal representation features constitute the correlation maximized representation space for text word embedding features and image CNN features. The generated representation features from cross-modal features are used to calculate similarities for cross-modal matching to search. The L2-norm is used to calculate the similarity presented as follows:

(10) $$\begin{array}{c} \text{sim}={\left\|S_{t}\left(x_{t}^{i};{\bar{\theta }}_{t}\right)-S_{v}\left(x_{v}^{j};{\bar{\theta }}_{v}\right)\right\|}_{2}. \end{array}$$

As presented in equation (6), the similarity calculation is included in the similarity loss. The similarity calculation is based on the optimized parameters ${\bar{\theta }}_{t}$ and ${\bar{\theta }}_{v}$ for appropriate results. The matching algorithm is shown in Algorithm 1.

Sorting and picking up the top *K* similarities are executed as the evaluation scope with the corresponding representation features. The corresponding content of the representation features in a list is returned according to the sorted top *K* similarities as the evaluation scope. The algorithm outputs cross-modal search results according to the query. The matching similarities are calculated based on the trained proposed method to obtain the most appropriate results.

## 4. Experiments and Analyses

Experiments on real-world datasets are conducted to verify the effectiveness of the proposed method on cross-modal search from social networks. The real-world datasets consisted of text-image pairs collected from Sina Weibo. Without loss of generality, the widely used Wikipedia [42] and NUS-WIDE [43] cross-modal datasets are also used to verify the effectiveness of the proposed method. In this section, the effects of changing empirical hyperparameter values and cross-modal search efficiency are shown and analyzed.

### 4.1. Experimental Setup

#### 4.1.1. Dataset Descriptions

The data collected from Sina Weibo of 4735 text-image pairs are about four security event topics from June 10, 2012, to September 7, 2016. There are 2866 text-image pairs of 10 categories in the Wikipedia dataset. In the NUS-WIDE dataset, there are 9000 text-image pairs in 350 categories. Following the traditional machine learning strategy, 70% of the data are used as a training set, while the rest are used as a test set for the two datasets.

#### 4.1.2. Evaluation Metrics

The mean average precision (MAP) for the top *K* and precision-scope curve are adopted as evaluation metrics to measure the performance of the proposed method. Following [33], MAP can be calculated as follows:

(11) $$\begin{array}{c} \text{AP}\left(Q\right)@K=\frac{\sum_{k=1}^{K}P\left(k\right)\delta \left(k\right)}{\sum_{k\prime =1}^{K}\delta \left(k^{\prime }\right)}, \end{array}$$

(12) $$\begin{array}{c} \text{MAP}@K=\frac{\sum_{q=1}^{Q}\text{AP}\left(Q\right)@K}{Q}. \end{array}$$

In equation (11), *Q* is the number of queries. *K* is the amount of the contents to be searched for results. The top *k* search precision is denoted as *P*(*k*), which is also adopted as a measure for the search results for the scope *K* presented as a precision-scope curve. The average precision is computed in equation (11) as a component of equation (12).

#### 4.1.3. Baselines

We compare the proposed CMSAL method with state-of-the-art methods on the Sina Weibo dataset and Wikipedia dataset. The selected methods are representative from classical applications to adversarial learning, such as JFSSL [44], CMDN [45], DCCA [12], ACMR [33], and CM-GAN [5].

#### 4.1.4. Parameter Learning Results and Analyses

**Figure 2:**
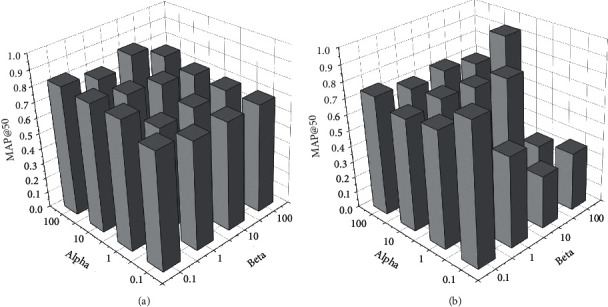
The empirical values impacting the cross-modal search based on the Sina Weibo dataset. (a) The empirical values impacting on txt2img search performance of MAP@50 and (b) the empirical values impacting on img2txt search performance of MAP@50.

**Figure 3:**
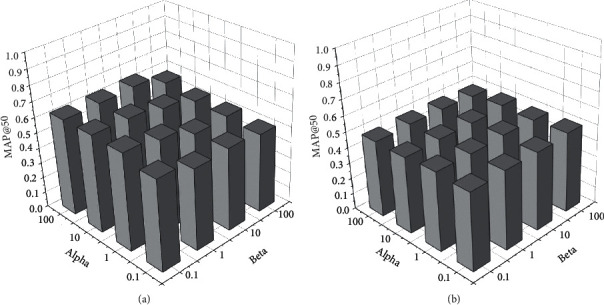
Empirical values impacting cross-modal search based on the Wikipedia dataset. (a) The empirical values impacting on txt2img search performance of MAP@50 and (b) the empirical values impacting on img2txt search performance of MAP@50.

**Figure 4:**
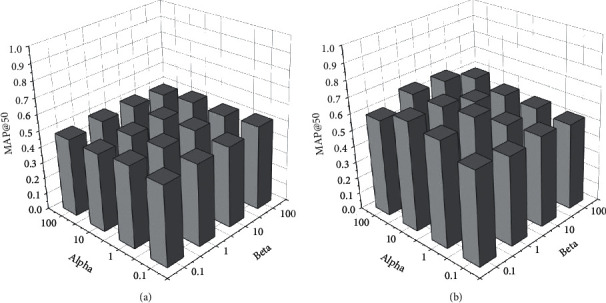
Empirical values impacting cross-modal search based on the NUS-WIDE dataset. (a) The empirical values impacting txt2img search performance of MAP@50 and (b) the empirical values impacting img2txt search performance of MAP@50.

We conduct an experiment to show the impact of the empirical values *α* and *β* in equation (6) for the searching performance, of which the results will provide a basis for setting the empirical values in return. MAP is used to evaluate the performance while the empirical values vary. The evaluations of the two datasets are presented in Figures 2–4. The empirical values of *alpha* and *beta* are the corresponding weight parameters for the label loss and the similarity loss.

As shown in Figure 2, we evaluate the top 50 search results based on computing MAP@50 for varying alpha and beta on the Sina Weibo dataset. The MAP@50 value shows different distributions with the common point that MAP@50 obtains a better situation when beta = 0.1. This means that the similarity loss requires a smaller weight value than alpha for a high MAP@50 evaluation. As shown in Figure 3, the effects of empirical values for searching performance on the Wikipedia dataset are smaller than those of the Sina Weibo dataset. Different from Figure 2, there is less fluctuation of MAP@50 varying the values of *alpha* and *beta*. The results presented in Figure 3 also provide a reference for the *alpha* and *beta*. Considering the situations of Figures 2 and 3, empirical values can be set with a group of suitable values for appropriate search results.

Figure 4 presents the empirical values impacting the cross-modal search based on the NUS-WIDE dataset. The numerical distribution is relatively flat, as in Figure 3 for the NUS-WIDE dataset. The results show that the dataset property has a direct impact on the empirical value assignment. Similar to the Wikipedia dataset, the semantics of the cross-modal information NUS-WIDE dataset are more obvious with less sparsity. Furthermore, the correspondence of cross-modal data in NUS-WIDE is clearer by using simple text content as a semantic label. Therefore, the empirical values impacting the image-to-text search performance of MAP@50 in the NUS-WIDE dataset are greater than those in the Wikipedia dataset.

The proposed method sets the empirical values of *alpha* and *beta* according to the dynamic evaluations as described. The learning process is inseparable from appropriate empirical values. We incorporated appropriate values of *alpha* = 1 and *beta* *=* 0.1 for image searches with text input and *alpha* = 0.1 and *beta* *=* 0.1 text searches with image input in both the Sina Weibo dataset and the Wikipedia dataset. According to Figure 4, *alpha* = 0.1 and *beta* *=* 100 for image searches with text input and *alpha* = 100 and *beta* *=* 10 for text searches with image input will be appropriate for the NUS-WIDE dataset.

### 4.2. Search Result Evaluations and Analyses

#### 4.2.1. MAP Evaluations and Analyses

Based on the appropriate values of *alpha* and *beta* for the learning process of the proposed CMSAL method, evaluations for search results are presented. In this section, we use MAP to show searching performances for the top 5, top 20, and top 50 results of CMSAL compared with the baseline methods. The evaluations on the Sina Weibo dataset are shown in Table 1, while those on the Wikipedia dataset are presented in Table 2. The evaluations on the NUS-WIDE dataset are presented in Table 3.

In Table 1, txt2img means entering a text query with the target topic to search from images with the same topics (img2txt means the reverse). As shown, the proposed CMSAL method outperforms the selected baseline methods. For CMSAL itself, the task of img2txt obtains better evaluations on MAP for the top 5 than those of the txt2img task. The reason for this situation is that original images contain abundant semantic information that will be extracted and represented appropriately. The extracted CNN features can preserve and present the valuable local semantics in detail completely, while semantic units of text are simpler for the same purpose. It will be more reliable when querying an image, with more semantic information for searching text with target semantics.

Table 2 shows MAP evaluations on the Wikipedia dataset. Compared with Table 1, the evaluation values on the Wikipedia dataset show lower values than those on the Sina Weibo dataset (in Table 1). The Sina Weibo dataset contains typical raw real-world data from various users, including casual written text and low-resolution images, which provide sparse cross-modal semantics. As expected, the results on the Sina Weibo dataset achieved higher evaluation values than the results on the Wikipedia dataset. The reason for this situation is that semantic features in the Sina Weibo dataset are relatively concentrated and prominent.

As shown in Table 3, the proposed CMSAL method outperforms the selected standard methods. MAP evaluations on the NUS-WIDE dataset are smaller than those on the Sina Weibo dataset. The main reason is that the characteristics of the NUS-WIDE dataset are different from those of the Sina Weibo and Wikipedia datasets. On the NUS-WIDE dataset, images are labeled with relatively simple text content, which clarifies the correspondence between text and images. In addition, in terms of image data quality, the NUS-WIDE dataset has simplified semantic information as public datasets. Therefore, the MAP evaluations of search results on the NUS-WIDE dataset are closer to those on Wikipedia datasets.

#### 4.2.2. Precision-Scope Evaluations and Analyses

**Figure 5:**
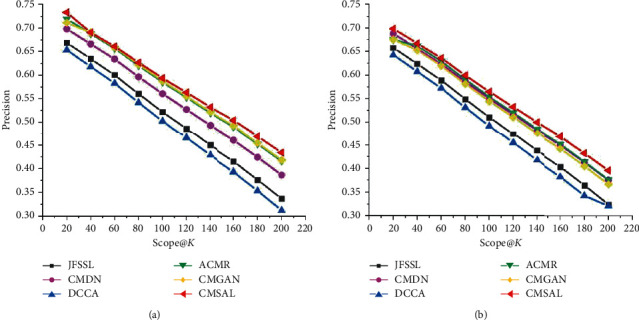
Precision-scope curves on the Sina Weibo dataset. (a) The precision-scope curve of the txt2img task and (b) the precision-scope curve of the img2txt task.

**Figure 6:**
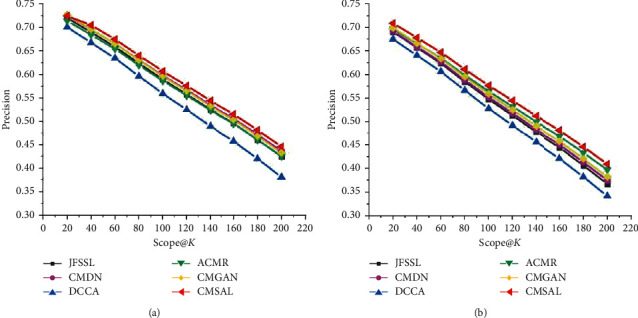
Precision-scope curves on the Wikipedia dataset. (a) The precision-scope curve of the txt2img task and (b) the precision-scope curve of the img2txt task.

**Figure 7:**
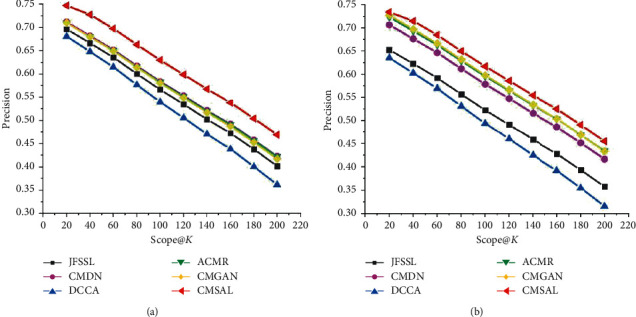
Precision-scope curves on the NUS-WIDE dataset. (a) The precision-scope curve of the txt2img task and (b) the precision-scope curve of the img2txt task.

As precision-scope curves are an indispensable form of evaluation for information search experiments, precision-scope curves of the proposed method CMSAL, and all the selected baseline methods. The experimental results on the Sina Weibo, Wikipedia, and NUS-WIDE datasets are shown in Figures 5–7.

As shown in Figures 5 and 6, the proposed CMSAL method shows a better performance than any of the other methods. In general, the measures of all the methods show similar trends with a small numerical gap. Similar to MAP evaluations, GAN-based methods achieve better performances than deep neural network- (DNN-) based methods which rely on targeted adversarial learning integrated with the advantages of DNNs. The classical DCCA method shows the worst values of the evaluation working in concert with MAP evaluations. The processing of nonlinear mapping and canonical correlation analysis learning is relatively independent for DCCA. However, the GAN-based method overcomes the disadvantages of traditional and DNN-based methods. The proposed method conducts appropriate representation feature generation to maximize correlations in adversarial learning. The results of the precision-scope curves demonstrate the effectiveness of the proposed method.

Figure 7 presents evaluations of precision-scope curves on the NUS-WIDE dataset for the tasks of searching for images from text input and searching for text from image input. As presented in Figure 7, the proposed CMSAL method outperforms other selected baseline methods. In addition, the precision-scope curves of CMSAL on the NUS-WIDE dataset outperform those on the Sina Weibo and Wikipedia datasets. The reason is that the cross-modal content in the NUS-WIDE dataset is simple and clear. As the semantic labels of images, the text has clear semantic features; thus, the tasks of text-to-image and image-to-text search show good computing properties in local semantic mining and matching for CMSAL.

## 5. Conclusions

In this paper, we propose a cross-modal search method for social network cross-modal data based on adversarial learning (CMSAL). The proposed method integrates self-attention based on adversarial learning to realize the cross-modal search for the social network. The method explores cross-modal semantic features from the perspective of global representations of images and texts for a specific topic. Through adversarial learning, the method reconstructs representations for cross-modal matching. The designed adversarial learning framework is effectively used to rank the search results. Experimental results validate the effectiveness of the proposed method.

## Figures and Tables

**Algorithm 1:**
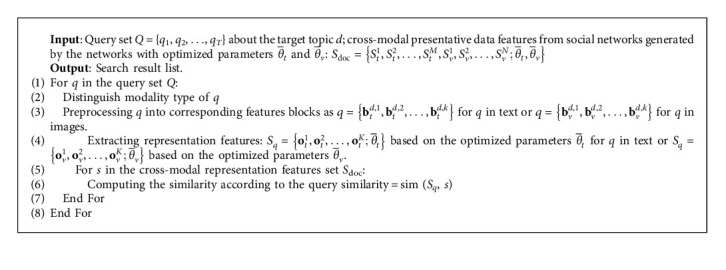
The matching algorithm of CMSAL.

**Table 1:** MAP evaluations on the Sina Weibo dataset.

		Traditional-based	DNN-based		GAN-based		
		JFSSL	CMDN	DCCA	ACMR	CMGAN	CMSAL
MAP@5	txt2img	0.6478	0.7183	0.3885	0.8653	0.8777	0.8898
	img2txt	0.5351	0.5814	0.3251	0.7133	0.7257	0.9481
	average	0.5915	0.6499	0.3568	0.7893	0.8017	0.919

MAP@20	txt2img	0.6128	0.6799	0.3583	0.8238	0.8362	0.8539
	img2txt	0.5181	0.5843	0.3239	0.7071	0.7195	0.9412
	average	0.5655	0.6321	0.3411	0.7655	0.7779	0.8975

MAP@50	txt2img	0.5197	0.5906	0.3213	0.7065	0.7189	0.8353
	img2txt	0.5282	0.5729	0.3199	0.6992	0.7116	0.9293
	average	0.5239	0.5817	0.3206	0.7029	0.7153	0.8823

**Table 2:** MAP evaluations on the Wikipedia dataset.

		Traditional-based	DNN-based		GAN-based		
		JFSSL	CMDN	DCCA	ACMR	CMGAN	CMSAL
MAP@5	txt2img	0.2685	0.4406	0.5094	0.6225	0.6629	0.6563
	img2txt	0.2151	0.3473	0.4125	0.4987	0.5391	0.5123
	average	0.2418	0.3940	0.4609	0.5606	0.6010	0.5843

MAP@20	txt2img	0.2831	0.4264	0.4895	0.6109	0.6513	0.6463
	img2txt	0.2209	0.3576	0.4102	0.4986	0.5390	0.5095
	average	0.252	0.392	0.4498	0.5548	0.5951	0.5779

MAP@50	txt2img	0.2543	0.4146	0.4624	0.5732	0.6136	0.6315
	img2txt	0.2178	0.3454	0.3956	0.4835	0.5239	0.5031
	average	0.2361	0.3800	0.4290	0.5284	0.5687	0.5673

**Table 3:** MAP evaluations on the NUS-WIDE dataset.

		Traditionally-based	DNN-based		GAN-based		
		JFSSL	CMDN	DCCA	ACMR	CMGAN	CMSAL
MAP@5	txt2img	0.2464	0.4187	0.5110	0.6397	0.6872	0.8023
	img2txt	0.2197	0.3313	0.4682	0.4838	0.4797	0.6329
	average	0.1337	0.3908	0.5877	0.6884	0.6248	0.7474

MAP@20	txt2img	0.2511	0.5469	0.4792	0.6077	0.6245	0.6796
	img2txt	0.2231	0.4965	0.5050	0.5284	0.5042	0.5887
	average	0.2450	0.3699	0.4012	0.6249	0.5314	0.6640

MAP@50	txt2img	0.3015	0.4011	0.5670	0.6500	0.6301	0.6798
	img2txt	0.1001	0.4700	0.3910	0.4801	0.5466	0.4836
	average	0.2587	0.4697	0.5035	0.6621	0.6653	0.7976

## Data Availability

The Sina Weibo dataset used to support the findings of this manuscript is collected with the help of Sina Weibo application interface from the website https://open.weibo.com/wiki/%E5%BE%AE%E5%8D%9AAPI?sudaref=www.baidu.com&display=0&retcode=6102. The Wikipedia dataset used to support the findings of this manuscript is taken from the website http://www.svcl.ucsd.edu/projects/crossmodal/. The NUS-WIDE dataset is taken from the website https://lms.comp.nus.edu.sg/wp-content/uploads/2019/research/nuswide/NUS-WIDE.html.
